# Exploring satisfaction level among outpatients regarding pharmacy facilities and services in the Kingdom of Saudi Arabia; a large regional analysis

**DOI:** 10.1371/journal.pone.0247912

**Published:** 2021-04-01

**Authors:** Nasser Hadal Alotaibi, Abdulaziz Ibrahim Alzarea, Ahmed Mohammed Alotaibi, Yusra Habib Khan, Tauqeer Hussain Mallhi, Khalid Saad Alharbi, Nabil K. Alruwaili, Abdullah S. Alanazi, Ahmed Hassan, Badriyah Shadid Alotaib

**Affiliations:** 1 Department of Clinical Pharmacy, College of Pharmacy, Jouf University, Sakaka, Al-Jouf, Kingdom of Saudi Arabia; 2 Department of Pharmacology, College of Pharmacy, Jouf University, Sakaka, Al-Jouf, Kingdom of Saudi Arabia; 3 Department of Pharmaceutics, College of Pharmacy, Jouf University, Sakaka, Al-Jouf, Kingdom of Saudi Arabia; 4 Health Sciences Research Unit, Jouf University, Jouf University, Sakaka, Al-Jouf, Kingdom of Saudi Arabia; 5 Department of Clinical Pharmacy, Faculty of Pharmacy, University of Sadat City (USC), Sadat City, Menoufia, Egypt; 6 Department of Pharmaceutical Sciences, College of pharmacy, Princess Nourah Bint Abdualrahman University, Riyadh, Saudi Arabia; Purdue University, UNITED STATES

## Abstract

**Background:**

Evaluation of patients`satisfaction towards pharmacy services is of utmost importance to ensure the quality of care. It helps in identifying domains requiring improvements to provide high quality pharmacy services to ensure the provision of enhanced pharmaceutical care. The current study aims to ascertain the extent of satisfaction towards pharmacy services among patients attending outpatient pharmacies in Kingdom of Saudi Arabia.

**Methods:**

A hospital-based cross-sectional study involving 746 patients attending outpatient pharmacies of various public hospitals was conducted from 01 January to 15 February 2020. Information on socio-demographic profile of the study subjects along with their satisfaction towards outpatient pharmacy was extracted by using a 23-items questionnaire. These questions were divided into two domains including 7 questions related to the pharmacy facilities (questions from 1F to 7F) and 8 questions for pharmacy services (questions from 1S to 8S), where F and S denotes facilities and services, respectively. The cumulative satisfaction score was estimated by a 5-item Likert scale with a maximum score of 5 for each item. The relationship between demographics and satisfaction scores was evaluated by using appropriate statistics.

**Results:**

There were 746 patients with male preponderance (58.8%). The overall satisfaction score was 2.97 ± 0.65. Satisfaction towards pharmacy services scored lower (mean score: 3.91 ± 0.77) than pharmacy facilities (mean score: 4.03 ± 0.66). Items related to patient`s counseling (3F, 2S, 3S, 6S) scored least during the analysis. Older patients (p = 0.006), male gender (p<0.001), Saudi nationality (0.035), patients attending primary care centers (p = 0.02), and patients with chronic illnesses were significantly associated with lower satisfaction score.

**Conclusion:**

This study reported that the satisfaction level of patients attending outpatient pharmacies was low and differed among various socio-demographic groups. Approximately one-half of the patients were not satisfied with outpatient pharmacy services. These findings underscore the dire need for managerial interventions including the hiring of trained professionals, onsite training of pharmacy staff, initiation of clinical or patient centered pharmacy services, evaluation of patient`s response towards the services and appropriate controlling measures, irrespective to the type of hospitals.

## Introduction

The concept of Good Pharmacy Practices (GPP) and Pharmaceutical Care (PC) revolves around optimal patient care with improved quality of life [1]. Since the quality assessment proportionate with patients`satisfaction, the quality standards of GPP and PC carry substantial importance in healthcare system. The quality of pharmaceutical services is assessed on three aspects including structure, process, and outcome. The structure includes assets and settings, while the process comprises activities, and outcomes include the effects of the care on the individual’s health [2]. Satisfaction of patients for pharmaceutical services reflects their preferences and expectations, and the realities of care. It is critical to understand the extent of dissatisfaction for pharmaceutical services [3]. The evaluation of satisfaction will help to identify the specific areas of the service in dire need of improvement and also enhance the positive changes in the current pharmaceutical services. This in turn will provide information to optimize services to ensure clients’ health outcomes by addressing their concerns and needs.

Pharmacists, in collaboration with other health professionals, have a responsibility to improve the quality of care among patients [4, 5]. Since the healthcare system has shifted from episodic care to population health management and from volume to value-based care, the role of pharmacists and their contribution has been emphasized in treatment and prevention of chronic diseases and their related complications [6, 7]. The outpatient hospital pharmacy (OPh) is a priority area of the pharmacy department in hospitals owing to a large number of visiting patients and the subsequent economic impact on the healthcare system. Appropriate use of human and financial resources in this area directly improves patient`s health outcomes [6].

Ministry of Health in Saudi Arabia has formulated a strategic plan for Pharmaceutical Care in 2012 comprising of five goals, seventeen initiatives, and eighty-three projects. This plan consists of a number of assessments and follow-up indicators of pharmacy and PC services. Of these, one of the major indicators is the patient satisfaction towards pharmacy services [8]. Though several studies have been conducted globally to ascertain the satisfaction of patients towards pharmacy services, there is a dearth of investigations in Kingdom of Saudi Arabia (KSA). The available literature have evaluated patient`s satisfaction towards community pharmacy [9], outpatient pharmacy services in specialized [10, 11] and tertiary care hospitals [12], or services provided in ambulatory care pharmacy [13] and pharmaceutical care [14]. To the best of our knowledge, none of the study has evaluated the patients’ satisfaction towards outpatient pharmacy services across different levels of care i.e. primary, secondary and tertiary care hospitals. Previously conducted studies were either confined to specialized pharmacy services, limited population, or concentrated to only one center, hereby precluding the generalizability of the findings across the regions. Besides, these studies do not provide an association between demographics and satisfaction levels of the patients. Moreover, there is no study that has evaluated the patients’ satisfaction in the northern region of KSA. In these contexts, the current study was aimed to ascertain the extent of satisfaction towards pharmacy services among patients attending the outpatient department of primary, secondary, and tertiary care hospitals and its relationship with socio-demographic characteristics.

## Methodology

### Ethics statement

Before initiation of the study, ethical approval was obtained by the institutional “Local Committee of Bioethics” (LCBE) (Reference no.: HAP-13-s-001). An informed consent was obtained from all study subjects. Prior to analysis, all the data were anonymized to protect the identification of study participants.

### Study site and population

**Fig 1:**
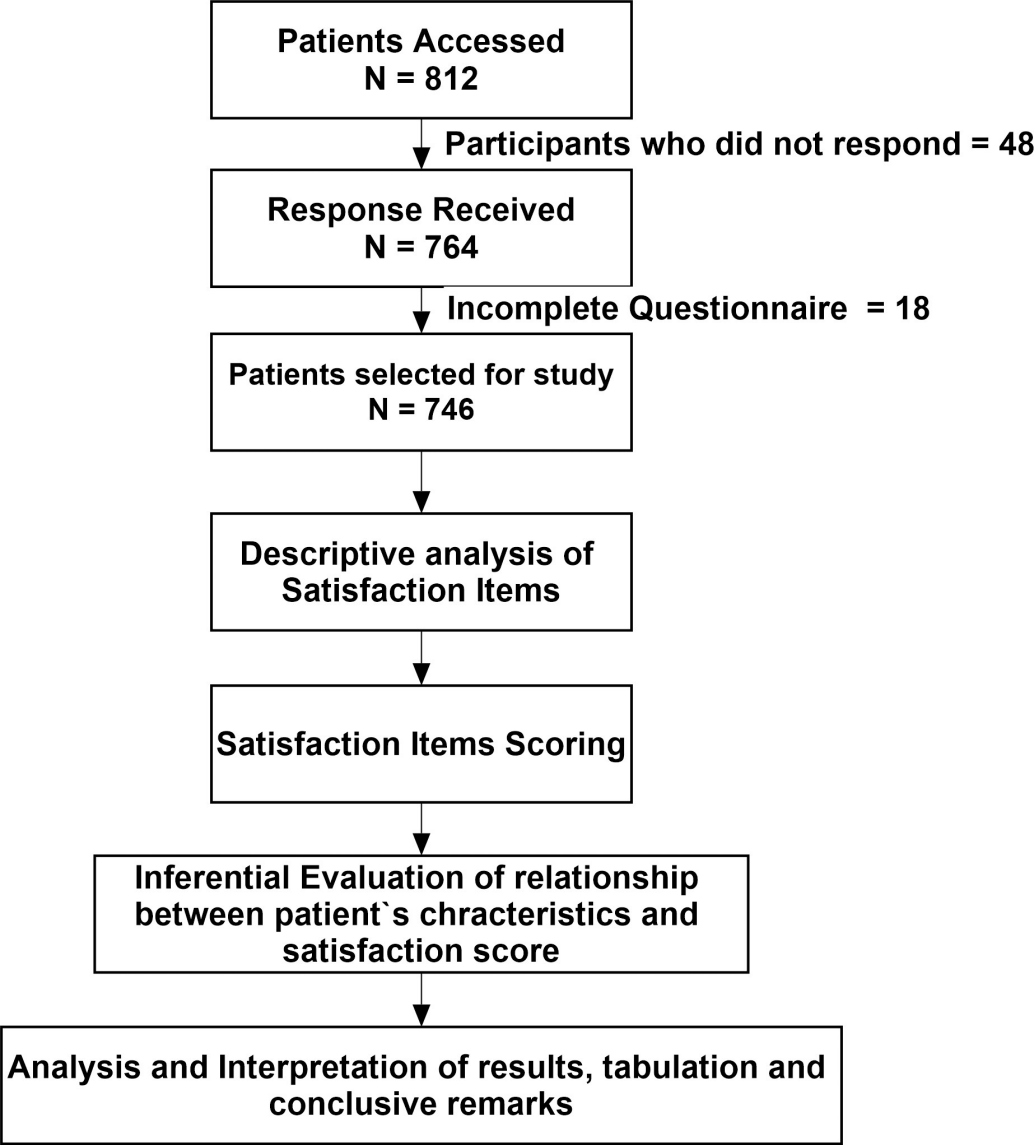
Study flow diagram.

This cross-sectional study (01 January– 15 February, 2020) was conducted among patients attending outpatient departments of primary (n = 6), secondary (n = 3) and tertiary hospitals (n = 3) in Al-Jouf region of Kingdom of Saudi Arabia (KSA). Al-Jouf is a Northern administrative region of KSA with estimated population of 0.5 million that shares border with Jordan. All the patients visiting outpatient pharmacy following their visits to the clinicians were eligible for this study. Patients who were not willing to participate or unable to communicate were excluded from the analysis. The methodological flow chart of the study is described in Fig 1.

### Sample size

Sample size was estimated through following assumptions: an accuracy of 5%, a confidence level of 95% and an expected satisfaction rate of 50%. After inclusion of 25% non-response rate, final sample size was calculated as 530 patients.

### Study instrument

A 23-items questionnaire, comprised of three sections, was developed under the opinions of experts from pharmacy professions (hospital pharmacists, clinical pharmacists, academicians, researchers in pharmaceutical sciences). The questionnaire was originally developed in English, and then translated into the national language (Arabic). Forward and backward translational accuracy was also ensured. The accuracy of translation was assessed by three language experts and inter-rater reliability was applied to find a proper translation (content validity). The contents of the instrument were pre-tested in a small and targeted sample of outpatients (n = 30) for assessment of clarity and comprehensibility. The alpha value of 0.795 indicated the reasonable level of reliability, adequacy, and internal consistency of the tool. Each section of the questionnaire included closed-ended questions with 5-items Likert scale. Section-I comprised of eight items related to the demographics while section-II consisted of seven questions evaluating the extent of patient satisfaction towards outpatient pharmacy facilities. This section evaluated satisfaction towards accessibility of pharmacy, the number of counters, privacy of counseling area, waiting area, waiting time, availability of prescribed, and other medications. Questions in this section were numbered from 1F to 7F, where “F” denotes facilities. Section-III had eight questions related to the satisfaction of patients towards pharmacy services provided to them during their visit to the hospitals. These questions evaluated the satisfaction of study participants regarding courtesy of pharmacy staff, guidance on medication use (dose, frequency), warnings and storage, time sufficiency for counseling, labeling, and overall experience with pharmacy services. All the questions in this section were numbered from 1S to 8S, where “S” denotes services. Likert scale was used to grade the satisfaction level among patients. The satisfaction of participants for each item was scored from 1 to 5. A score of 1 indicated “Strongly dissatisfied’, 2 denoted “dissatisfied”, 3 indicated “Neither satisfied nor dissatisfied”, 4 represented “Satisfied”, and 5 denoted “strongly satisfied”. The maximum satisfaction score was 5 for each item. The average score of all participants was estimated against each item (sum of satisfaction scores against item/total number of respondents). The mean satisfaction score for pharmacy facilities and services was also calculated. The satisfaction score of patients was further categorized into satisfied (score ≥ 3) and dissatisfied (score < 3). The scoring system adopted in the current study has been previously used in several research investigations [15, 16].

### Data collection

Using convenience sampling technique, patients from primary, secondary, and tertiary care centers were contacted for interview. The participation of patients in this study was voluntarily. Authors collected the data on three days of the week (Sunday to Tuesday) during 2 hours’ time slot (10 AM– 12 PM). The purpose of the study was explained to respondents and those who agreed to participate were given the questionnaire. A written informed consent was obtained from each patient. Participants were encouraged to complete the questionnaire. Face-to-face interviews were also conducted for participants with low education level or shortage of time. To ensure appropriate responses and quality of data, all the interviews were conducted according to the feasibility of the participants. All the questionnaires were checked for completeness of response. At the end, data were transferred to a Microsoft spreadsheet for cleaning purposes.

### Statistical analysis

All the data were analyzed by SPSS version 22.0. A significance level of 0.05 was used throughout. All continuous variables were reported as mean (standard deviation) or median (25%–75% interquartile range), while categorical variables were reported as counts (n) and proportions (%). Chi-square test or student-t test was used to compare categorical and continuous data between the groups. One way analysis of variance (ANOVA) was used to compare the satisfaction score if there were more than two categories. Tukey`s HSD was used for post-hoc analysis. Effect size of the relationship between score and demographics were evaluated through Eta squared.

## Results

Out of 812 respondents, a total of 764 responses were received (response rate: 94.1%), and 746 were included for the analysis after excluding non-responsive (n = 48) and incomplete (n = 18) forms. More than half of the patients had age less than 36 years with male preponderance (58.8%). Most of the participants had bachelor degree (58.4%) followed by secondary school certificate (29.2%). Saudi was the most common nationality (85.7%) in the current study. More than half of the responses were collected from tertiary care hospital (54.4%). About one fourth of patients sought medical care for acute conditions while others had chronic reasons to visit the hospitals. Major proportion of the patients visited the hospitals to refill their prescriptions. The demographics of the study participants enrolled in the current study are described in Table 1.

**Table 1:** Socio-demographic characteristics of study participants.

Variable	Number of Patients N = 746	Percentage (%)
**Age group**		
18–25 years	180	24.1
26–35 years	256	34.3
36–45 years	194	26
46–55 years	88	11.8
>55 years	28	3.8
**Gender**		
Male	439	58.8
Female	307	41.2
**Education level**		
No formal education	28	3.8
Primary school	20	2.7
Secondary school	218	29.2
Bachelor	436	58.4
Master	30	4
Diploma*	14	1.9
**Nationalities**		
Saudi	639	85.7
Non-Saudi	107	14.3
Egyptian	48	6.4
Sudanese	18	2.4
Syrian	17	2.3
Indian	10	1.3
Pakistani	7	0.9
Palestinian	2	0.3
Tunisia	3	0.4
Jordanian	2	0.3
**Type of Hospital**		
Primary	255	34.2
Secondary	85	11.4
Tertiary	406	54.4
**Occupation**		
Government employees^1^	258	34.6
Private employees^2^	110	14.7
Business^3^	37	5
Student	116	15.5
Labor/other^4^	117	15.7
Unemployed^5^	108	14.5
**Type of disease**		
Acute disease	188	25.2
Chronic disease	558	74.8
**Reason to Visit the Hospital**		
Medication refill^6^	602	80.7
Consultation^7^	144	19.3

*Two year certified program for professional training

^1^serving any government sector

^2^serving any private sector

^3^own small or large scale business

^4^working on daily wages (electrician, plumber, carpenter)

^5^respondents who are not doing any job

^6^patients who visited the hospitals to refill their prescriptions or formal check-up of previous acute or chronic disease

^7^patients who visited the hospital for detailed checkup by the consultant

Cumulative satisfaction score of study participants was 2.97 ± 0.65 out of 5 (Table 2). Current analysis provided stratified score for pharmacy facilities and services. Mean satisfaction score towards pharmacy facilities was 4.03 ± 0.659. Two facilities related questions (3F: privacy for the counseling area and 7F: overall experience on the availability of drugs in the pharmacy) scored low near to the dissatisfaction range as compared to other items. It is pertinent to mention that most of the patients were satisfied (score: 4.37 ± 1.07) with the availability of the drugs during their current visit to the pharmacy, indicating they received medications as prescribed to them. However, their overall experience towards the availability of the drugs in the hospital was dissatisfactory (score: 3.46 ± 1.33), where 187 patients reported that they are not satisfied with the availability of drugs in pharmacy. These findings indicate that patients are experiencing drug shortage during their visits to the hospital. However, neutral responses (n = 186) for item 7F must be considered while interpreting the results. Overall satisfaction of the patients towards pharmacy services was less than that of pharmacy facilities. Satisfaction score towards pharmacy services was lowest for item 3S (3.08 ± 1.64) followed by 6S (3.11 ± 1.63) and 2S (3.17 ± 1.60). These items were related to the history taking by the pharmacist, provision of information on drug warnings and storage.

**Table 2:** The proportions and mean satisfaction scores of respondents towards pharmacy facilities and services.

S.N	Item	Strongly Dissatisfied	Dissatisfied	Neither satisfied nor dissatisfied	Satisfied	Strongly Satisfied	Satisfaction score
Domain I*: Satisfaction related to Pharmacy Facilities							
1F	Are you satisfied with the pharmacy access in hospital?	51 (6.8)	31 (4.2)	76 (10.2)	129 (17.3)	459 (61.5)	4.23 ± 1.2
2F	Are you satisfied with the number of counters in the pharmacy?	26 (3.5)	27 (3.6)	64 (8.6)	172 (23.1)	457 (61.3)	4.35 ± 1.02
3F	Are you satisfied with the privacy for the counseling area? (privacy, noise free, separate)	112 (15)	212 (28.4)	91 (12.2)	170 (22.8)	161 (21.6)	3.08 ± 1.41
4F	Are you satisfied with the comfort of waiting area located in the pharmacy?	25 (3.4)	25 (3.4)	75 (10.1)	151 (20.2)	470 (63)	4.36 ± 1.02
5F	Are you satisfied with the waiting time for dispensing in the pharmacy?	12 (1.6)	33 (4.4)	91 (12.2)	164 (22)	446 (59.8)	4.34 ± 0.96
6F	Are you satisfied that during your current visit you receive the medications from the pharmacy exactly according to the prescription? (Did you receive all the medicines prescribed to you during this visit?)	32 (4.3)	33 (4.4)	51 (6.8)	138 (18.5)	446 (59.8)	4.37 ± 1.07
7F	Considering your previous visits in the hospital, are you satisfied with the availability of medicines in the pharmacy? (Are all the medicines that prescribed to you by the doctors always available in the pharmacy whenever you visit the hospital?)	75 (10.1)	112 (15)	186 (24.9)	142 (19)	231 (31)	3.46 ± 1.33
Mean Satisfaction towards Pharmacy Facilities**		**4.03 ± 0.66**					
Domain II*: Satisfaction related to Pharmacy Services							
		**Strongly Dissatisfied**	**Dissatisfied**	**Neither satisfied nor dissatisfied**	**Satisfied**	**Strongly Satisfied**	
1S	Are you satisfied with the courtesy of pharmacist or staff (pharmacist was courteous, supportive and helpful to me)	1 (0.1)	4 (0.5)	36 (4.8)	111 (14.9)	594 (79.6)	4.73 ± 0.58
2S	Are you satisfied with the history taking by pharmacist? (Pharmacist or staff asked you about your health and medication history before dispensing the medication)	176 (23.6)	117 (15.7)	109 (14.6)	89 (11.9)	255 (34.2)	3.17 ± 1.60
3S	Are you satisfied with necessary instructions and warnings about your medications (side effects, drug-drug interactions, food and drug interactions), especially for medications received for the 1st time?	209 (28)	105 (14.1)	88 (11.8)	107 (14.3)	237 (31.8)	3.08 ± 1.64
4S	Are you satisfied with the sufficiency of time given to you for counseling? (was counseling time enough to answer all your questions)	18 (2.4)	34 (4.6)	108 (14.5)	159 (21.3)	427 (57.2)	4.26 ± 1.02
5S	Are you satisfied with the labeling of the medicine? (labeling was clear and easy to understand)	16 (2.1)	19 (2.5)	85 (11.4)	140 (18.8)	486 (65.1)	4.42 ± 0.94
6S	Are you satisfied with the storage information for medicine provided to you? (storage information was clear and understandable)	195 (26.1)	123 (16.5)	82 (11)	99 (13.3)	247 (33.1)	3.11 ± 1.63
7S	Are you satisfied with all other information related to the use of medicine provided to you by pharmacist/staff? (dose, frequency)	42 (5.6)	38 (5.1)	59 (7.9)	116 (15.5)	491 (65.8)	4.31 ± 1.17
8S	Are you satisfied with the pharmacy services you received during your visit? (your overall satisfaction with pharmacy services)	12 (1.6)	33 (4.4)	104 (13.9)	217 (29.1)	380 (50.9)	4.23 ± 0.96
Mean Satisfaction towards Pharmacy Services***		**3.91 ± 0.77**					
**Cumulative Satisfaction Score towards Pharmacy Services and Facilities**^**€**^		**2.97 ± 0.65**					

*Under domain I and II data was presented as Frequency with percentage (%)

**Mean satisfaction score of 7 items (1F-7F) evaluating the respondent`s satisfaction towards pharmacy facilities

***Mean satisfaction score of 8 (1S-7S) items evaluating the respondent`s satisfaction towards pharmacy services

^**€**^ Cumulative satisfaction score refers to average satisfaction scores of both domains

Table 3 demonstrates relationship between socio-demographics and satisfaction score towards pharmacy facilities and services among study participants. It shows that patients aged between 26 to 35 years, female gender, patients attending tertiary care hospitals, labor profession and those with acute diseases were significantly associated with higher cumulative satisfaction score. All the other demographic parameters showed meaningless inferences during the analysis. Subgroup comparison of each domain revealed substantial association of age, gender, occupation and type of disease with satisfaction towards pharmacy facilities (Domain I). On the other hand, gender, type of hospital, occupation and type of disease were significantly associated with patient`s satisfaction towards pharmacy services (Domain II).

**Table 3:** Relationship between socio-demographics and satisfaction score among study participants.

	Domain I: Satisfaction towards pharmacy facilities: Items (1F to 7F)		Domain II: Satisfaction towards pharmacy Services: Items (1S to 8S)		Cumulative Satisfactions score towards pharmacy facilities and services	
Variable	Satisfaction Score (Mean ± SD) N = 746	P-values	Satisfaction Score (Mean ± SD) N = 746	P-values	Satisfaction Score (Mean ± SD) N = 746	P-values
**Age group**		**<0.001**		0.100		**0.006**
18–25 years	3.95 ± 0.69*		3.85 ± 0.71		2.89 ± 0.64*	
26–35 years	4.17 ± 0.59*		3.99 ± 0.82		3.07 ± 0.66*	
36–45 years	4.01 ± 0.71		3.91 ± 0.79		2.95 ± 0.69	
46–55 years	3.93 ± 0.56*		3.93 ± 0.72		2.93 ± 0.56	
>55 years	3.77 ± 0.79*		3.63 ± 0.67		2.69 ± 0.65*	
**Gender**		**< 0.001**		**< 0.001**		**< 0.001**
Male	2.82 ± 0.62		3.73 ± 0.73		2.82 ± 0.62	
Female	3.18 ± 0.65		4.18 ± 0.75		3.18 + 0.65	
**Education level**		0.422		0.079		0.129
No formal education	4.18 ± 0.46		3.96 ± 0.66		3.06 ± 0.48	
Primary school	4.00 ± 0.69		3.99 ± 0.83		2.99 ± 0.68	
Secondary school	4.04 ± 0.66		3.93 ± 0.71		2.98 ± 0.62	
Bachelor	4.02 ± 0.68		3.91 ± 0.81		2.96 ± 0.62	
Master	4.04 ± 0.65		4.00 ± 0.73		3.02 ± 0.59	
Diploma	3.70 ± 0.37		3.29 ± 0.69		2.49 ± 0.43	
**Saudi versus Non-Saudi**		0.551		0.195		0.242
Saudi	4.02 ± 0.66		4.01 ± 0.69		2.96 ± 0.66	
Non-Saudi	4.06 ± 0.64		3.90 ± 0.79		3.03 ± 0.60	
**Nationalities**		0.311		0.159		0.165
Saudis	4.02 ±0.66		3.90 ±0.78		2.96 ± 0.66	
Egyptian	3.95 ±0.55		3.89 ±0.58		2.92 ± 0.48	
Sudanese	4.41 ±0.53		4.35 ±0.62		3.37 ± 0.55	
Syrian	4.19 ±0.66		4.26 ±0.99		3.23 ± 0.79	
Indian	4.00 ±0.53		3.60 ±0.41		2.79 ± 0.38	
Pakistani	3.94 ±0.87		3.86 ±0.49		2.90 ± 0.61	
Palestinian	4.07 ±0.50		3.81 ±0.27		2.93 ± 0.38	
Tunisia	3.95 ±1.57		4.25 ±0.88		3.11 ± 1.19	
Jordanian	3.43 ± 1.01		3.75 ±1.24		2.60 ± 1.13	
**Type of Hospital**		0.618		**0.001**		**0.02**
Primary	4.00 ± 0.62		3.76 ± 0.80*		2.87 ± 0.65*	
Secondary	4.08 ± 0.67		3.99 ± 0.86		3.03 ± 0.70	
Tertiary	4.03 ± 0.68		3.99 ± 0.72*		3.02 ± 0.67*	
**Occupation**		**0.002**		**< 0.001**		**< 0.001**
Government employees	3.98 ± 0.70*		3.81 ± 0.83*		2.89 ± 0.70*	
Private employees	4.01 ± 0.62		3.89 ± 0.80		2.95 ± 0.65*	
Business	3.93 ± 0.63		3.83 ± 0.67		2.88 ± 0.57	
Students	3.93 ± 0.63*		3.81 ± 0.65*		2.87 ± 0.57*	
Labor/other	4.06 ± 0.70		4.06 ± 0.74*		3.22 ± 0.57	
Unemployed	4.26 ± 0.56*		4.18 ± 0.70*		3.06 ± 0.66*	
**Type of disease**		**< 0.001**		**< 0.001**		**< 0.001**
Acute disease	3.22 ± 0.68		4.20 ± 0.79		3.22 ± 0.68	
Chronic disease	2.88 ± 0.62		3.82 ± 0.74		2.88 ± 0.63	
**Reason to Visit the Hospital**		0.627		0.709		0.672
Medication refill	2.96 ± 0.65		3.91 ± 0.77		2.96 ± 0.65	
Consultation	2.99 ± 0.66		3.94 ± 0.77		2.99 ± 0.66	

*Tukey`s HSD posthoc analysis demonstrating significant variables among multiple comparisons

Effect size (Cohen`s d): age group = 0.02, type of hospital = 0.01, occupation = 0.03, Gender = 0.0730, Saudi vs Non-Saudi = 0.0018, Type of disease = 0.0505

All the patients were stratified into two categories (satisfied or dissatisfied) on the basis of their satisfaction score. Based on cutoff value of < 3 for dissatisfaction, 363 patients were dissatisfied with the outpatient pharmacy facilities and services (Table 4). Subgroup analysis revealed that patient age, gender, education level, nationality, occupation and type of disease were substantially associated with the satisfaction status. Patients aged between 26 to 35 years were more satisfied, while a large proportion of male gender reported significant dissatisfaction with outpatient pharmacy services and facilities. Non-Saudis were more satisfied than Saudis (P = 0.035). Similar to the prior analysis, students and those with chronic illness were not satisfied with the outpatient pharmacy facilities and services.

**Table 4:** Factors associated with satisfaction status for pharmacy facilities and services among outpatients.

	Satisfied patients N = 383	Dissatisfied patients N = 363	P values
**Age group**			**0.018**
18–25 years	81 (21.1)	99 (27.3)	
26–35 years	151 (39.4)	105 (28.9)	
36–45 years	98 (25.6)	96 (26.4)	
46–55 years	43 (11.2)	45 (12.4)	
>55 years	10 (2.6)	18 (5.0)	
**Gender**			**<0.001**
Male	182 (47.5)	257 (70.8)	
Female	201 (52.5)	106 (29.2)	
**Education level**			**0.027**
No Formal Education	15 (3.9)	13 (3.6)	
Primary School	12 (3.1)	8 (2.2)	
Secondary School	119 (31.1)	99 (27.3)	
Bachelor	221 (57.7)	215 (59.2)	
Master	15 (3.9)	15 (4.1)	
Diploma	1 (0.3)	13 (3.6)	
**Saudi versus Non-Saudi**			**0.035**
Saudi	318 (83)	321 (88.4)	
Non-Saudi	65 (17)	42 (11.6)	
**Nationalities**			0.152
Saudis	318 (83.0)	321 (88.4)	
Egyptian	24 (6.3)	24 (6.6)	
Sudanese	15 (3.9)	3 (0.8)	
Syrian	12 (3.1)	5 (1.4)	
Indian	5 (1.3)	5 (1.5)	
Pakistani	5 (1.3)	2 (0.6)	
Palestinian	1 (0.3)	1 (0.3)	
Tunisia	2 (0.5)	1 (0.3)	
Jordanian	1 (0.3)	1 (0.3)	
**Type of Hospital**			0.134
Primary	118 (30.8)	137 (37.7)	
Secondary	45 (11.7)	40 (11.0)	
Tertiary	220 (57.4)	186 (51.2)	
**Occupation**			**0.001**
Government Employees	116 (30.3)	142 (39.1)	
Private employees	57 (14.9)	53 (14.6)	
Business	19 (5.0)	18 (5.0)	
Students	51 (13.3)	65 (17.9)	
Labor/other	75 (19.6)	33 (9.1)	
Unemployed	65 (17.0)	52 (14.3)	
**Type of disease**			**<0.001**
Acute disease	125 (32.6)	63 (17.4)	
Chronic disease	258 (67.4)	300 (82.6)	
**Reason to Visit the Hospital**			0.260
Consultation	80 (20.9)	64 (17.6)	
Medication Refill	303 (79.1)	299 (82.4)	

P values were calculated using chi-square test or Fischer Exact test, where appropriate

Data is presented in Frequency with proportion (%)

## Discussion

To the best of our knowledge, current study is the first to explore the extent of patients`satisfaction towards outpatient pharmacy facilities and services, and its association with socio-demographic characteristics among diverse group of patients attending different types of hospitals in the northern region of KSA. Previous investigations conducted in KSA are accompanied by several limitations including specialized hospital study site [11], few questions in study instrument [12], monocentric study design and small sample size [10]. Moreover, there is dearth of investigations evaluating the relationship of patient`s demographics and satisfaction score. Alturki and Khan elaborated the association between demographics and satisfaction levels but their findings cannot be implicated generally as this study was conducted in Ear, Nose and Throat (ENT) specialized hospital [11].

The ministry of health in Saudi Arabia has set priority goals to implement the Strategic Plan of General Administration of Pharmaceutical Care. According to this plan, patients`satisfaction is considered a pivotal goal to ensure the optimal pharmaceutical care services. In this context, present study carries several important implications for healthcare policy makers. The results of this study demonstrated low patient satisfaction (mean score: 2.97 ± 0.65) with the pharmacy encounters. These findings are in contrast with the other studies conducted in KSA where patients attending tertiary care hospitals reported good level of satisfaction towards pharmacy services [10, 11]. Ahmad et al. (2016) in their cross-sectional survey indicated good satisfaction among patients attending armed forces hospital. In addition, authors found high level of dissatisfaction (score: 1.891) towards the attitude of pharmacy service providers. However, it is pertinent to mention that this study evaluated patients`satisfaction towards the availability and accessibility of drugs in the pharmacy [10]. Since the availability of drugs in military hospitals remains prioritized by the government, it might be a possible reason that patients had higher satisfaction score in their study. On the other hand, Alturki & Khan (2013) estimated satisfaction in a specialized ENT hospital by using self-administered 15-items questionnaire and found good consumer satisfaction [10]. It must be noted that majority of the specialized hospitals remain equipped with state of art facilities, well qualified staff, and better control system which may attribute to the better satisfaction score in their study as compared to our results. These studies vary in methodological aspects and study population which precludes the direct comparison of our findings with them. However, low level of satisfaction in the current study is comparable to international surveys [1, 17–19]. The global criteria to measure the patient satisfaction varies markedly. Different cultural settings have different needs or expectation that affect the overall satisfaction with the health care services. Moreover, regional development and facilities must be considered while interpreting the results. Al-Jouf province differs by cultural norms as it is a least developed region and has limited healthcare facilities such as expert physicians and pharmacists as compared to the other provinces of KSA which may contribute to the low level of satisfaction among patients.

Out of two major domains, pharmacy services scored less (score: 3.91 ± 0.77) as compared to pharmacy facilities (score: 4.03 ± 0.659), indicating unsatisfactory performance of pharmacy departments in the hospitals. Of pharmacy facilities, privacy of the counseling area (item no. 3F) scored less as compared to other six items. These findings are consistent with the results of Ahmad et al. (2016) where lack of privacy for counseling area was primarily associated with the low level of satisfaction among patients [10]. Lack of counseling areas in pharmacies along with inappropriate location of such areas have been demonstrated as a predictor of patients dissatisfaction in various international studies [1, 20]. Since patients counseling is mainstay in disease management, these findings necessitate a dire need of administrative measures to develop appropriate and private areas for counseling to achieve optimal outcomes.

It is worthy to note that three items (2S, 3S, 6S) from domain related to pharmacy services scored lowest in the current study. Alarmingly, all these items were related to the patient counseling. Most of the study participants reported that they were not satisfied with history taking practice by the pharmacists (Score: 3.17 ± 1.60), provision of instructions for side effects or interactions (Score: 3.08 ± 1.64) and dissemination of information on storage conditions (Score: 3.11 ± 1.63). Drug related problems (DRPs) including side effects, interactions, and low or high dose pose substantial risks of toxicity, suboptimal effect or poor adherence. Pharmacists are poised to play an important role in improving medication management during transitions of care. They have potential to educate patients about the importance of continued therapy and adherence, and also to resolve any uncertainties that patients may have regarding their medications. The low level of satisfaction towards counseling services has been reported in recent studies among patients attending outpatient hospital or community pharmacies [1, 21, 22]. Dissatisfaction of patients for counseling services in the current study might be attributed to less number of pharmacists and pharmacy staff in the hospitals, increased workload, professional in-competencies or lack of continuous education for pharmacy service providers. These findings underscore the dire need of implementation of pharmaceutical care services in outpatient pharmacy department with appropriately equipped human resources. Appropriate number of staff and effective time management could help pharmacists manage their traditional and clinical roles. This aspect is of paramount importance to pharmacy human resource as proper staffing would ensure better performance that could result in increased satisfaction from service and translate into optimal patients`outcomes. The relationship of workload on pharmacists and provision of counselling services has been well discussed in the literature [23, 24].

Current study elaborates substantial associations of patients’ demographics with satisfaction levels. Younger patients, female gender, those attending tertiary care hospitals were significantly associated with higher satisfaction score. Unlike other studies [1, 10], young patients within age category of 26 to 35 years were more satisfied. Most of the study population was comprised of young patients and it might be a possible reason of high satisfaction score among young adults. Moreover, various other studies conducted in KSA where study population majorly comprised of young patients showed no relationship of patients`satisfaction with age [10, 12]. In another study conducted in Spain, age was not significantly associated with satisfaction [4]. Recently, Aziz et al. (2018) found higher satisfaction score among patients with age ranging from 26 to 30 years [22]. The higher level of satisfaction among young adults in the current study might be attributed to lower prevalence of chronic diseases than older patients. Better health conditions in younger patients may also contribute to higher satisfaction as previous studies have demonstrated direct relationship between self-reported health, young age and high satisfaction. It has been well documented that younger patients have good self-reported health and have better satisfaction to healthcare services [25]. Various studies describe complex relationship between patient age and satisfaction scores suggesting that age must be considered as a non-linear factor during the interpretation of satisfaction towards the healthcare facilities [26]. Such discrepancies and ambiguities in existing literature call for more investigations to establish the impact of age on patients’ satisfaction. Since current study has small number of elderly patients which preclude us to draw a firm conclusion on association of patient’s age and satisfaction, future studies must be carried out by incorporating appropriate sampling technique according to the age of the patients.

Unemployed patients had significantly higher satisfaction score than government/private sector employees and students. Though the score was highest among laborers but it did not differ significantly from other occupations in post-hoc analysis. These results coincides with the findings of another study conducted in Ethiopia where authors showed good satisfaction among farmers and patients without any job [10]. It must be noted that satisfaction score was also high among patients without any formal education. It can be assumed that these patients are either laborer or unemployed. The higher level of satisfaction among these patients might be attributed to the low awareness on standard pharmacy services. Moreover, these patients may have lower expectations from healthcare system than educated or employed patients. Similar findings were observed by Kamei et al. (2001) reporting higher satisfaction score in unemployed patients [27]. However, our results are in contrast to a study conducted in ENT hospital of KSA where education levels positively correlated with satisfaction score [11]. Such discrepancies across the literature require further studies for elaboration of the relationship between education levels and satisfaction among patients.

Tertiary care hospitals are equipped with better healthcare facilities than primary or secondary care centers. It might be a possible contributor to the higher satisfaction among patients attending tertiary care centers. The number of patients attending primary and tertiary care facilities was substantially higher in the current study. Though the satisfaction score among patients attending secondary care settings was highest but post-hoc analysis failed to demonstrate any inferential relationship. Similar findings have been observed in another study conducted in KSA, where patients attending both primary and tertiary centers demonstrated good satisfaction [26]. In contrast, our findings indicate the lowest satisfaction score for primary healthcare centers. The low level of patients’ satisfaction in primary centers might be attributed to the limited number healthcare staff, diagnostics and management facilities and availability of drugs.

Patients with acute illnesses scored higher satisfaction than those with chronic diseases. These findings are in contrast with previous reports where patients with chronic illnesses were more likely to be satisfied with counseling and pharmaceutical services compared to those with acute illnesses [28, 29]. Another study conducted on Saudi population showed no association of patients`satisfaction with the type of illness [23]. Hamann et al. investigated the patients`desire to participate in medical decisions by using Autonomy Preference Index (API) and found that patients with acute conditions have similar API score than those with chronic illnesses [30]. There is scarcity of data exploring the relationship of type of illness and patients`satisfaction towards the pharmacy services. However, our findings corroborate the results of other studies where patients with acute conditions had higher satisfaction score towards hospital services as compared to those with chronic diseases [31, 32]. The higher satisfaction score among patients with acute diseases might be attributed to young age and their optimistic approach towards their outcomes. Tennakon and Zoysa (2014) also associated higher satisfaction among acute patients with their optimistic approach [32]. Since most of the respondents in our study were young adults who scored higher satisfaction, the association of acute illnesses with higher satisfaction might be confounded by the patients’ age. In addition, this study contains few elderly patients which further bias the findings due to less representation of chronic illnesses. However, frequent hospital encounters and deteriorating health conditions in chronic patients can be possible factors for low satisfaction. Keeping in view the wide disparity in the available literature and contradicting findings from our study on the relationship of satisfaction and type of illness, a firm conclusion could not be drawn based on presumptive factors. Our analysis demonstrates the dire need of more studies to affirm these findings.

This study determines several demographic features in association with patients’ satisfaction towards outpatient pharmacy services. Patients were also stratified into satisfied or dissatisfied according to their satisfaction scores. This analysis demonstrated that Saudis were significantly less satisfied than non-Saudis (*p* = 0.035). It might be attributed to the fewer expectations of expatriates towards healthcare facilities in KSA. These findings are in concordance with another study conducted in ENT hospital in KSA [11].

Low level of satisfaction in the current study might be associated with limited number of pharmacists, lower standards of pharmacy services and less attention of regional health departments on pharmacy standards and practice. Hiring more trained professionals, initiating clinical pharmacy or patient centered services, onsite trainings and appropriate controlling measures will aid to improve the pharmacy services in the region. Findings of present study warrant dire needs of aggressive maneuvers from regional and national health administrators.

### Study limitation and strengths

Despite several important findings originated from the analysis, results of the current study must be interpreted in the light of the following shortcomings. This study does not address specific pharmacy services such as educational interventions for diabetic, hypertensive and dialytic patients. Moreover, study instrument does not have thorough assessment on pharmaceutical care services. Supplementing the study tool with specific pharmaceutical care questions will aid to stratify the extent of patient`s satisfaction towards traditional as well as extended pharmacy services. Most of the study participants were young which hinder generalizability of the findings in settings dealing chronic, elderly and pediatric patients. Similarly, data was collected from one region of KSA which limits the generalization of the findings across the country. In addition, patients’ health condition was not assessed in the study which might be a confounding factor during interpretation of the results. Last but not least, criterion used to classify patients into satisfied and dissatisfied was quite strict but it complies with the KSA`s 2030 vision of world class health facilities.

Nevertheless, despite notable limitations, the current study is strengthened by the first report on satisfaction towards outpatient pharmacy department among diverse group of patients attending different healthcare facilities in KSA. Moreover, large sample size, heterogeneous population and evaluation of both facilities and services of pharmacies are other strengths. Additionally, vigorous analysis of results enables future studies to compare their findings with ours. Results of the present study will facilitate healthcare administrators to tailor the policies in poorly performing areas of pharmacy department.

## Conclusions

The present study explicitly demonstrates the low level of satisfaction among outpatients towards the pharmacy facilities and services of primary, secondary or tertiary care hospitals. Subgroup analysis indicated the association of age, gender, education level, nationality, occupation and type of disease with patient`s satisfaction. Since patients who are more satisfied with pharmacy services are more likely to be compliant with various aspects of treatment and to return to providers for additional care, special attention should be paid to optimize the satisfaction of clients attending outpatient pharmacies. There is dire need to investigate the extent of patient-pharmacist interaction in the public hospitals of KSA. These findings necessitate the need of national level surveys to ascertain the relationship of patients with pharmacy staff or services, so that targeted measures could be designed and implemented in the future.

## Supporting information

S1 File (PDF)

S2 File (PDF)

S1 Data (SAV)
