# Supplementary material for: Exploring satisfaction level among outpatients regarding pharmacy facilities and services in the Kingdom of Saudi Arabia; a large regional analysis
Source: PLoS One. 2021 Apr 1;16(4):e0247912. doi: 10.1371/journal.pone.0247912 (PMC8016244; doi:10.1371/journal.pone.0247912)
Supplement: S1 File — (PDF) [file pone.0247912.s001.pdf]

<u>DATA COLLECTION FORM</u>

Exploring Satisfaction Level among Outpatients regarding Pharmacy Facilities and Services in Kingdom of Saudi Arabia; A large Regional Analysis

<u>Section I: Demographics</u>

Nationality:…………………………….

Gender: Male ☐ Female ☐

Type of Hospital: Tertiary☐ Secondary☐ primary☐

Age: (18-25) ☐ (26-35) ☐ (36-45) ☐ (46-55) ☐ (More than55) ☐

Occupation: Governmental Employee ☐ Privet Employee ☐ Businessman ☐ Student ☐

Nonemployee ☐ Others…………

Education level: No formal education ☐ Primary school ☐ Secondary school ☐ Bachelor ☐ Master ☐ Others/Labor: ________________

Type of disease: Chronic disease ☐ Others: ……………………….

Visit reason: Consultation ☐ Medication refill ☐

S.N	Item	Strongly Dissatisfied	Dissatisfied	Neither satisfied nor dissatisfied	Satisfied	Strongly Satisfied	Satisfaction score
Section I: Satisfaction towards Pharmacy Facilities							
1	Are you satisfied with the pharmacy access in hospital?						
2	Are you satisfied with the number of counters in the pharmacy?						
3	Are you satisfied with the privacy for the counseling area? (privacy, noise free, separate)						
4	Are you satisfied with the comfort of waiting area located in the pharmacy?						
5	Are you satisfied with the waiting time for dispensing in the pharmacy?						
6	Are you satisfied that during your current visit you received the medications from the pharmacy exactly according to the prescription? (Did you receive all the medicines prescribed to you during this visit?)						
7	Considering your previous visits in the hospital, are you satisfied with the availability of medicines in the pharmacy? (Are all the medicines that prescribed to you by the doctors always available in the pharmacy whenever you visit the hospital?)						
Section II: Satisfaction towards Pharmacy Services							
8	Are you satisfied with the courtesy of pharmacist or staff (pharmacist was courteous, supportive and helpful to me)						
9	Are you satisfied with the history taking by pharmacist? (Pharmacist or staff asked you about your health and medication history before dispensing the medication)						
10	Are you satisfied with necessary instructions and warnings about your medications (side effects, drug-drug interactions, food and drug interactions), especially for medications received for the 1st time?						
11	Are you satisfied with the sufficiency of time given to you for counseling? (was counseling time enough to answer all your questions)						
12	Are you satisfied with the labeling of the medicine? (labeling was clear and easy to understand)						
13	Are you satisfied with the storage information for						

	medicine provided to you? (storage information was clear and understandable)						
14	Are you satisfied with all other information related to the use of medicine provided to you by pharmacist/staff? (dose, frequency)						
15	Are you satisfied with the pharmacy services you received during your visit? (your overall satisfaction with pharmacy services)						

<u>Thank you for your time an input</u>
