# Supplementary material for: Exploring satisfaction level among outpatients regarding pharmacy facilities and services in the Kingdom of Saudi Arabia; a large regional analysis
Source: PLoS One. 2021 Apr 1;16(4):e0247912. doi: 10.1371/journal.pone.0247912 (PMC8016244; doi:10.1371/journal.pone.0247912)
Supplement: S2 File — (PDF) [file pone.0247912.s002.pdf]

استبانة "معرفة مستوى الرضى بين مرضى العيادات الخارجية عن التسهيلات والخدمات الصيدلانية المقدمة بالمملكة العربية السعودية"

الأخ /الأخت : هذا البحث عبارة عن مشروع بحثي لكلية **الصيدلة بجامعة الجوف** والغرض منه هو السعي في تحسين الخدمات الصيدلانية المقدمة للمرضى. وهذه الاستبيان بحثي فقط ولن يذكر فيه أسماء المرضى.

معلومات عامه :-

الجنسية:------------------------ **الجنس:** ☐ ذكر ☐ أنثي

نوع مؤسسة الرعاية الصحية: ☐ رجعي ☐ ثانوي ☐ لي

العمر: ☐ (٢٥-١/ ☐ (٣٥-٢٦ ☐ (٤٥-٣٦ ☐ (٥٥-٤٦ ☐ كثر من ٥٥ سنة)

المهنة: ☐ ظف حكومي ☐ ظف قطاع خاص ☐ جل عمل ☐ الب ☐ أعمل

أخرى:--

المستوى التعليمي: ☐ ير متعلم ☐ تدائي ☐ توسط ☐ نوي ☐ جامعي واكثر

نوعية المرض: ☐ مرض مزمن ☐ أخرى: ------------------------------------

الغرض من زيارة المركز / المستشفى : ☐ تابعة ☐ تشارة ☐ لاعادة صرف الأدوية

تسلسل		غير راضي بشدة	غير راضي	الى حد ما	راضي	راضي بشدة
**القسم الاول: الرضى تجاه التسهيلات بالصيدلية**						
1	مدى رضاك عن سهولة الوصول الى الصيدلية					
2	مدى رضاك عن عدد نوافذ صرف الادوية بالصيدلية					
3	مدى رضاك عن خصوصية مكان الاستشارة بالصيدلية (خاص، لايوجد ازعاج، منعزل)					
4	مدى رضاك عن مستوى الراحة بمنطقة انتظار صرف الدواء بالصيدلية					
5	مدى رضاك عن فترة وقت الانتظار لصرف الادوية بالصيدلية					
6	مدى رضاك عن صرف الصيدلية لأدويتك المكتوبه لك بالوصفه خلال زيارتك الحالية (هل استلمت جميع الادوية المكتوبه لك بالوصفه لك خلال هذه الزياره؟)					
7	من خلال زياراتك السابقه للمستشفى ما مدى رضاك العام عن توفر الأدوية بالصيدلية (هل الادوية الصروفة لك من الطبيب متوفره دائما بالصيدلية خلال زياراتك؟)					
**القسم الثاني: الرضى عن خدمات الصيدلية**						
8	مدى رضاك عن تعامل الصيدلي معك (هل كان الصيدلي متساعدا معك و مهذبا و داعما؟)					
49	مدى رضاك عن قيام الصيدلي بسؤالك عن تاريخك المرضي والادوية التي تسخدمها (هل سألك الصيدلي عن وضعك و ادويتك التي كنت تسنخدمها قبل صرف ادويتك؟)					
10	مدى رضاك عن التعليمات و التحذيرات الضرورية لادويتك					

	المصروفة (المضار الجانبية، تفاعلات الادوية مع بعضها، تفاعلات الادوية مع الاغذية) وخاصة الادوية التي صرفت لك لاول مرة					
11	مدى رضاك عن الزمن الذي قضاه الصيدلي معك بالاستشاره (هل كان وقت الاستشاره مناسب للإجابة على جميع جميع استفساراتك؟)					
12	مدى رضاك عن الملصق لادويتك (يحتوي على معلومات واضحه ومفهومه)					
13	مدى رضاك عن معلومات طريق التخزين للادوية المصروفة لك (ملعومات التخزين واضحة وسهلة الفهم)					
14	مدى رضاك عن المعلومات الاخرى التي قدمت لك من الصيدلي والتي تتعلق بكيفية استخدام الدواء (الجرعه، مرات اخذ الدواء)					
15	مدى رضاك عن الخدمات المقدمه لك من الصيدلية خلال زيارتك (رضاك العام عن خدمات الصيدلية)					
